# Supplementary material for: Structures of autoinhibited and polymerized forms of CARD9 reveal mechanisms of CARD9 and CARD11 activation
Source: Nat Commun. 2019 Jul 11;10:3070. doi: 10.1038/s41467-019-10953-z (PMC6624267; doi:10.1038/s41467-019-10953-z)
Supplement: Supplementary file 1 — Supplementary Information [file 41467_2019_10953_MOESM1_ESM.pdf]

Supplementary Information

Structures of autoinhibited and polymerized forms of CARD9 reveal mechanisms of CARD9 and CARD11 activation

M.J. Holliday et al.

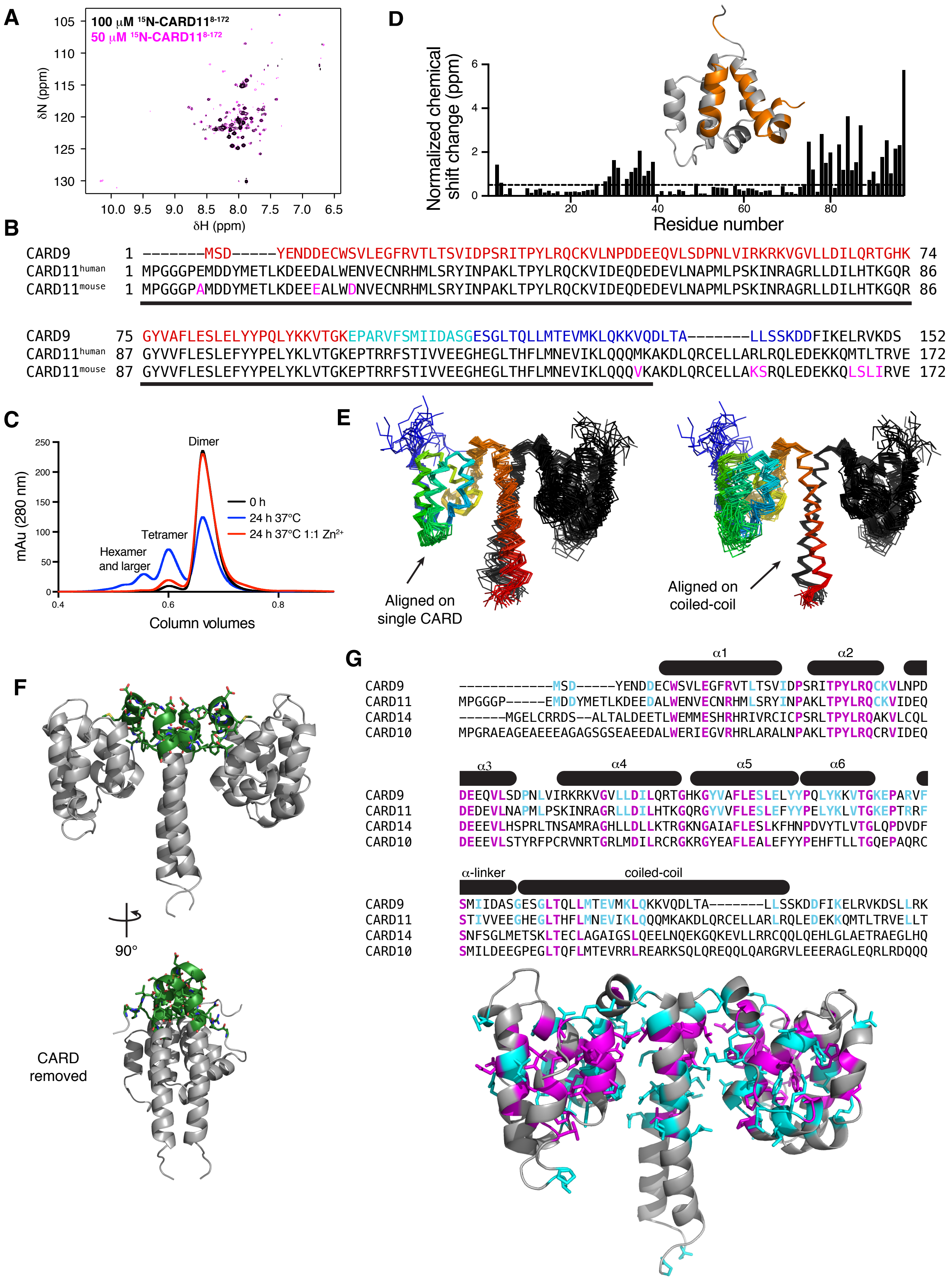
A
100 μM 15N-CARD11 8-172
50 μM 15N-CARD11 8-172
δN (ppm)
δH (ppm)
D
Normalized chemical shift change (ppm)
Residue number
B
CARD9 1 -------MSD-----YENDDECWSVLEGFRVTLTSVIDPSRITPYLRQCKVLNPDDEEQVLSDPNLVIRKRKVGVLLDILQRTGHK 74
CARD11human 1 MPGGGPEMDDYMETLKDEEDALWENVECNRHMLSRYINPAKLTPYLRQCKVIDEQDEDEVLNAPMLPSKINRAGRLLDILHTKGQR 86
CARD11mouse 1 MPGGGPAMDDYMETLKDEEEALWDNVECNRHMLSRYINPAKLTPYLRQCKVIDEQDEDEVLNAPMLPSKINRAGRLLDILHTKGQR 86
CARD9 75 GYVAFLESLELYYPQLYKKVTGKEPARVFSMIIDASGESGLTQLLMTEVMKLQKKVQDLTA-------LLSSKDDFIKELRVKDS 152
CARD11human 87 GYVVFLESLEFYYPELYKLVTGKEPTRRFSTIVVEEGHEGLTHFLMNEVIKLQQQMKAKDLQRCELLARLRQLEDEKKQMTLTRVE 172
CARD11mouse 87 GYVVFLESLEFYYPELYKLVTGKEPTRRFSTIVVEEGHEGLTHFLMNEVIKLQQQVKAKDLQRCELLAKSRQLEDEKKQLSLIRVE 172
C
Dimer
Tetramer
Hexamer and larger
0 h
24 h 37°C
24 h 37°C 1:1 Zn2+
mAu (280 nm)
Column volumes
E
Aligned on single CARD
Aligned on coiled-coil
F
90°
CARD removed
G
α1
α2
CARD9 ------------MSD-----YENDDECWSVLEGFRVTLTSVIDPSRITPYLRQCKVLNPD
CARD11 MPGGGP-----EMDDYMETLKDEEDALWENVECNRHMLSRYINPAKLTPYLRQCKVIDEQ
CARD14 ------MGELCRRDS--ALTALDEETLWEMMESHRHRIVRCICPSRLTPYLRQAKVLCQL
CARD10 MPGRAEAGEAEEEAGAGSGSEAEEDALWERIEGVRHRLARALNPAKLTPYLRQCRVIDEQ
α3
α4
α5
α6
CARD9 DEEQVLSDPNLVIRKRKVGVLLDILQRTGHKGYVAFLESLELYYPQLYKKVTGKEPARVF
CARD11 DEDEVLNAPMLPSKINRAGRLLDILHTKGQRGYVVFLESLEFYYPELYKLVTGKEPTRRF
CARD14 DEEEVLHSPRLTNSAMRAGHLLDLLKTRGKNGAIAFLESLKFHNPDVYTLVTGLQPDVDF
CARD10 DEEEVLSTYRFPCRVNRTGRLMDILRCRGKRGYEAFLEALEFYYPEHFTLLTGQEPAQRC
α-linker
coiled-coil
CARD9 SMIIDASGESGLTQLLMTEVMKLQKKVQDLTA-------LLSSKDDFIKELRVKDSLLRK
CARD11 STIVVEEGHEGLTHFLMNEVIKLQQQMKAKDLQRCELLARLRQLEDEKKQMTLTRVELLT
CARD14 SNFSGLMETSKLTECLAGAIGSLQEELNQEKGQKEVLLRRCQQLQEHLGLAETRAEGLHQ
CARD10 SMILDEEGPEGLTQFLMTEVRRLREARKSQLQREQQLQARGRVLEEERAGLEQRLRDQQQ

Supplementary Figure 1. Details of the CARD9$^{2\text{-}152}$ structure and sequence alignments within the protein family, related to Figures 1 and 3 (A) ^{15}N-TROSY spectra of CARD11$^{8\text{-}172}$ collected at 50 μM (magenta) or 100 μM (black) (B) Sequence alignment of the N-terminus of human CARD9, human CARD11, and mouse CARD11. Residues comprising the CARD, linker, and coiled-coil sub-domains of CARD9 are colored in red, cyan, and blue, as in Figure 1A. Differences between the human and mouse CARD11 are shown in magenta. The regions of the proteins that were swapped in the chimeric constructs (Figure 3) are indicated by the black line. (C) Superdex 200 gel filtration chromatography UV_{280} traces for 1 mM CARD$^{2\text{-}152}$ just after purification and concentration (black), after 16 hours at 37°C (blue), and after 16 hours at 37°C with equimolar Zn^{2+} (red). (D) Normalized backbone amide chemical shift changes between CARD9$^{2\text{-}97}$ and the CARD9$^{2\text{-}152}$ dimer. Dotted line is at 0.5 ppm; residues with chemical shift changes greater than 0.5 ppm are plotted in orange on the NMR structure of CARD9$^{2\text{-}97}$ (PDBID 6E26). (E) CARD9$^{2\text{-}142}$ dimer structure aligned on the backbone of one of the CARDs (top) or the central coiled-coil (bottom). (F) CARD9$^{2\text{-}142}$ with residues 100-118, which are homologous to residues 112-130 previously identified as the LATCH in CARD11, highlighted in green. (G) Sequence alignment of the four CARD proteins, CARD9, CARD10, CARD11, CARD14. Residues identically conserved among all four homologues are colored magenta both in the sequence and on the CARD9$^{2\text{-}142}$ NMR structure. Residues identically conserved only between CARD9 and CARD11 are colored in cyan both in the sequence and on the CARD9 NMR structure. Residues comprising the 6 CARD α-helices as well as the linker and coiled-coil α-helices are indicated by thick bars.

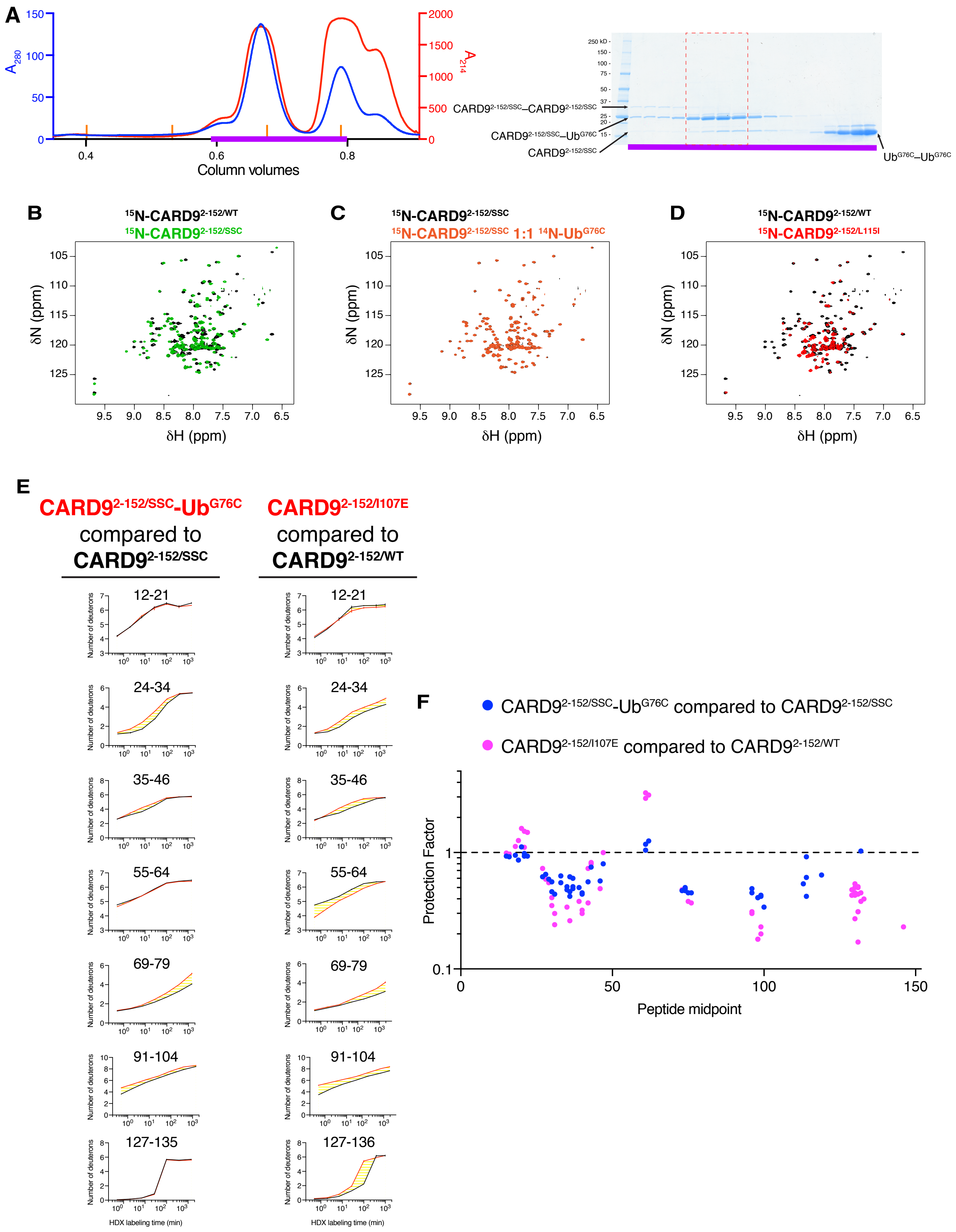

A
A280
A214
Column volumes
CARD9^2-152/SSC–CARD9^2-152/SSC
CARD9^2-152/SSC–Ub^G76C
CARD9^2-152/SSC
Ub^G76C–Ub^G76C
B
15N-CARD9^2-152/WT
15N-CARD9^2-152/SSC
C
15N-CARD9^2-152/SSC
15N-CARD9^2-152/SSC 1:1 14N-Ub^G76C
D
15N-CARD9^2-152/WT
15N-CARD9^2-152/L115I
δN (ppm)
δH (ppm)
E
CARD9^2-152/SSC-Ub^G76C compared to CARD9^2-152/SSC
CARD9^2-152/I107E compared to CARD9^2-152/WT
12-21
24-34
35-46
55-64
69-79
91-104
127-135
127-136
Number of deuterons
HDX labeling time (min)
F
CARD9^2-152/SSC-Ub^G76C compared to CARD9^2-152/SSC
CARD9^2-152/I107E compared to CARD9^2-152/WT
Protection Factor
Peptide midpoint

Supplementary Figure 2. Generation and characterization of chemically ubiquitinated CARD9$^{2\text{-}152/SSC}$, related to Figure 4 (A) Representative Superdex 200 size exclusion chromatography trace for conjugation of CARD9$^{2\text{-}152/SSC}$ to Ub^{G76C}. Sample was prepared as described in Experimental Methods. Absorbance at 280 and 214 nm are plotted in blue and red, respectively. Ub^{G76C} lacks a tryptophan and so exhibits a much lower extinction coefficient at 280 nm than CARD9$^{2\text{-}152/SSC}$. Fractions indicated by the purple line were run on an SDS-PAGE gel and stained with Coomassie brilliant blue (right). Species present in the fractions are indicated. Representative fractions that were pooled for final sample are boxed in red. Minor band above the Ub^{G76C}—Ub^{G76C} and CARD9$^{2\text{-}152/SSC}$—Ub^{G76C} bands reflects a minor population of Ub^{G76C} for which the 6xHis tag was not removed during purification. (B) ^{15}N-TROSYs of CARD9$^{2\text{-}152/WT}$ (black) and CARD9$^{2\text{-}152/SSC}$ (green) under identical conditions. (C) ^{15}N-TROSYs of CARD9$^{2\text{-}152/SSC}$ alone (black) or in the presence of equimolar unconjugated Ub^{G76C} (orange). (D) ^{15}N-TROSYs of CARD9$^{2\text{-}152/WT}$ (black) and CARD9$^{2\text{-}152/L115I}$ (red). (E) Representative deuterium exchange plots for peptides that could be identified in experiments both comparing CARD9$^{2\text{-}152/SSC}$—Ub^{G76C} to CARD9$^{2\text{-}152/SSC}$ (left column) and CARD9$^{2\text{-}152/I107E}$ to CARD9$^{2\text{-}152/WT}$ (right column). Data for peptide 24-34 on the left are the same as shown in Figure 4G. With the exception of the final peptide pair, the identical peptide is shown for both experiments. Equivalent sets of peptides were not identified towards the N-terminus of CARD9$^{2\text{-}152}$ in the two experiments, and so peptides 127-135 and 127-136 are shown. (F) Global protection factors for CARD9$^{2\text{-}152/SSC}$—Ub^{G76C} compared to CARD9$^{2\text{-}152/SSC}$ (blue) and CARD9$^{2\text{-}152/I107E}$ compared to CARD9$^{2\text{-}152/WT}$ (magenta). Each dot represents the mid-point of a single identified peptide. Dotted line indicates a protection factor of 1, indicating no change upon conjugation. Source data are provided in Source Data file.

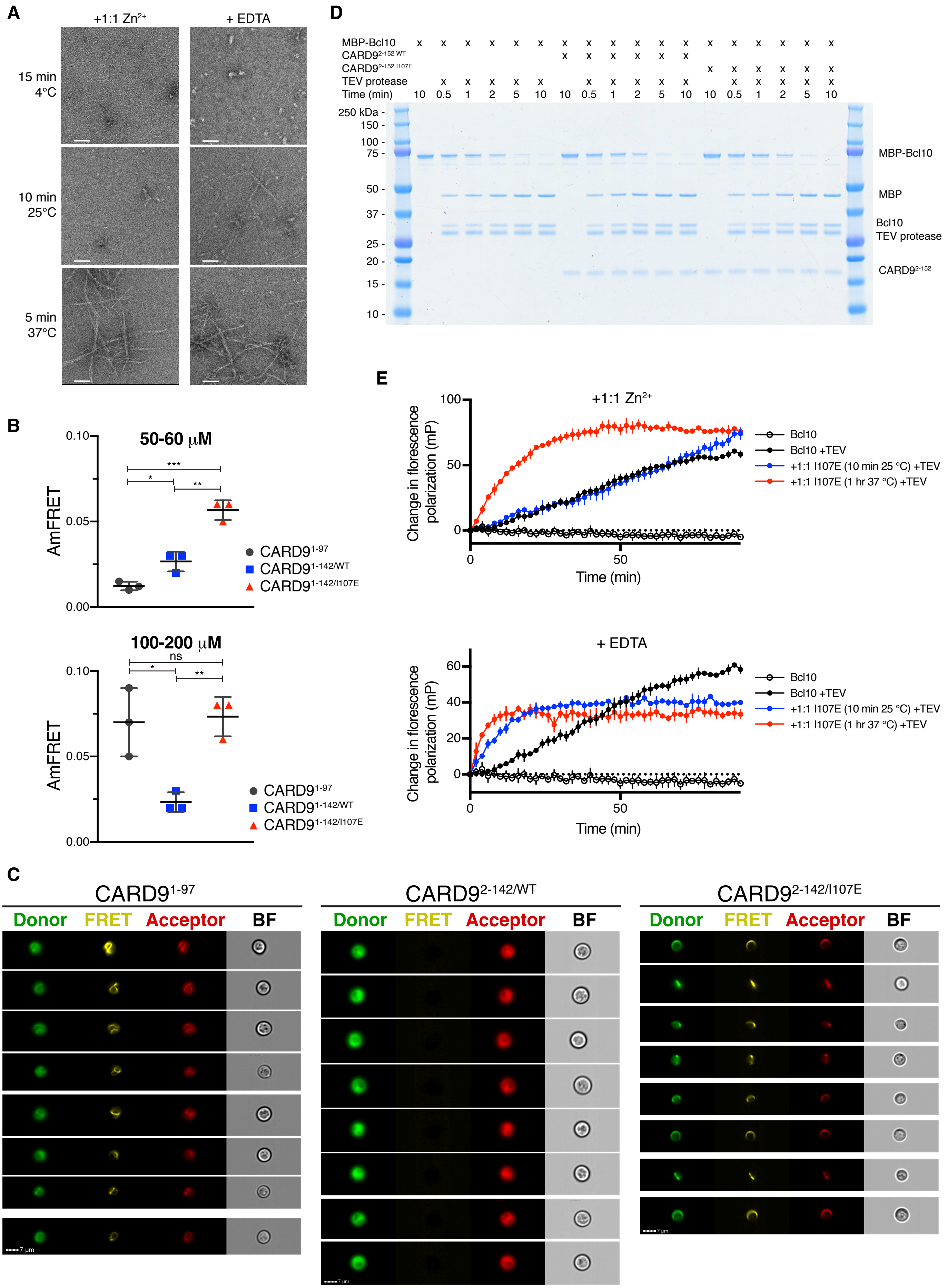

A
+1:1 Zn2+
+ EDTA
15 min 4°C
10 min 25°C
5 min 37°C
B
50-60 μM
100-200 μM
AmFRET
0.10
0.05
0.00
***
*
**
ns
CARD9 1-97
CARD9 1-142/WT
CARD9 1-142/I107E
C
CARD9 1-97
CARD9 2-142/WT
CARD9 2-142/I107E
Donor
FRET
Acceptor
BF
7 μm
D
MBP-Bcl10
CARD9 2-152 WT
CARD9 2-152 I107E
TEV protease
Time (min)
10 0.5 1 2 5 10 10 0.5 1 2 5 10 10 0.5 1 2 5 10
250 kDa
150
100
75
50
37
25
20
15
10
MBP-Bcl10
MBP
Bcl10
TEV protease
CARD9 2-152
E
+1:1 Zn2+
+ EDTA
Change in florescence polarization (mP)
Time (min)
Bcl10
Bcl10 +TEV
+1:1 I107E (10 min 25 °C) +TEV
+1:1 I107E (1 hr 37 °C) +TEV

Supplementary Figure 3. CARD9 helical assembly and Bcl10 nucleation, related to Figure 5 (A) NS-EM micrographs of CARD9$^{2\text{-}152/I107E}$ after incubation at 0.5 mM for the indicated times at the indicated temperatures. The stock protein was prepared conjugated 1:1 to Zn^{2+}; At time t=0, 1 mM EDTA was added to the samples shown on the right to strip the Zn^{2+} away from CARD9$^{2\text{-}152/I107E}$. All samples were diluted to 50 μM just before grid preparation. Scale bars are 200 nm (B) Binned data from three independent replicates of the DAmFRET experiment depicted in Figure 5B. Data were binned at 50-60 μM (top) or 100-200 μM (bottom). P-values from a two-tailed t-test are indicated by ns (>0.05), * (< 0.05), ** (< 0.005), or *** (< 0.0005). (C) Representative images of imaging flow cytometry data for CARD9$^{1\text{-}97}$, CARD9$^{1\text{-}142/WT}$, and CARD9$^{1\text{-}142/I107E}$ related to Figure 5B. Cells were gated in a window of >100 μM protein concentration. Images represent the signal (from left to right) of mEos3.1 donor, FRET, mEos3.1 acceptor, and bright field. CARD9$^{1\text{-}97}$ and CARD9$^{1\text{-}142/I107E}$ exhibit polymers in the FRET channel, while CARD9$^{1\text{-}142/WT}$ exhibits a complete lack of FRET signal. (D) Representative MBP-Bcl10 cleavage time course after addition of TEV to MBP-Bcl10 alone (left), MBP-BCl10 with 1:1 CARD9$^{2\text{-}152/WT}$ (center), or MBP-BCl10 with 1:1 CARD9$^{2\text{-}152/I107E}$ (right). CARD9$^{2\text{-}152}$ samples were prepared by removing Zn^{2+} and incubating at 0.5 mM for 10 min at 25°C as in Figure 5B. (E) Bcl10 nucleation assay. CARD9$^{2\text{-}152/I107E}$ was incubated at 0.5 mM either at 25°C for 10 min or at 37°C for 60 min, either as prepared with 1:1 Zn^{2+} (top) or with addition of 1 mM EDTA (bottom). These solutions were then added at a 1:1 concentration to MBP-Bcl10 (2 μM final concentrations) as indicated and TEV protease was added at time t=0. As shown in panel A, after 10 minutes at 25°C, CARD9$^{2\text{-}152/I107E}$ filaments only form when Zn^{2+} is removed and, correspondingly, of the CARD9$^{2\text{-}152/I107E}$ samples prepared at 25°C, only the +EDTA sample accelerates Bcl10 polymerization. After 1 hour at 37°C, CARD9$^{2\text{-}152/I107E}$ filaments form both apo and Zn^{2+}-bound; as expected if helical assembly is required for Bcl10 nucleation, both of these samples are capable of accelerating Bcl10 polymerization. Vertical bars represent the standard deviation of three technical replicates. All source data are provided in Source Data file.

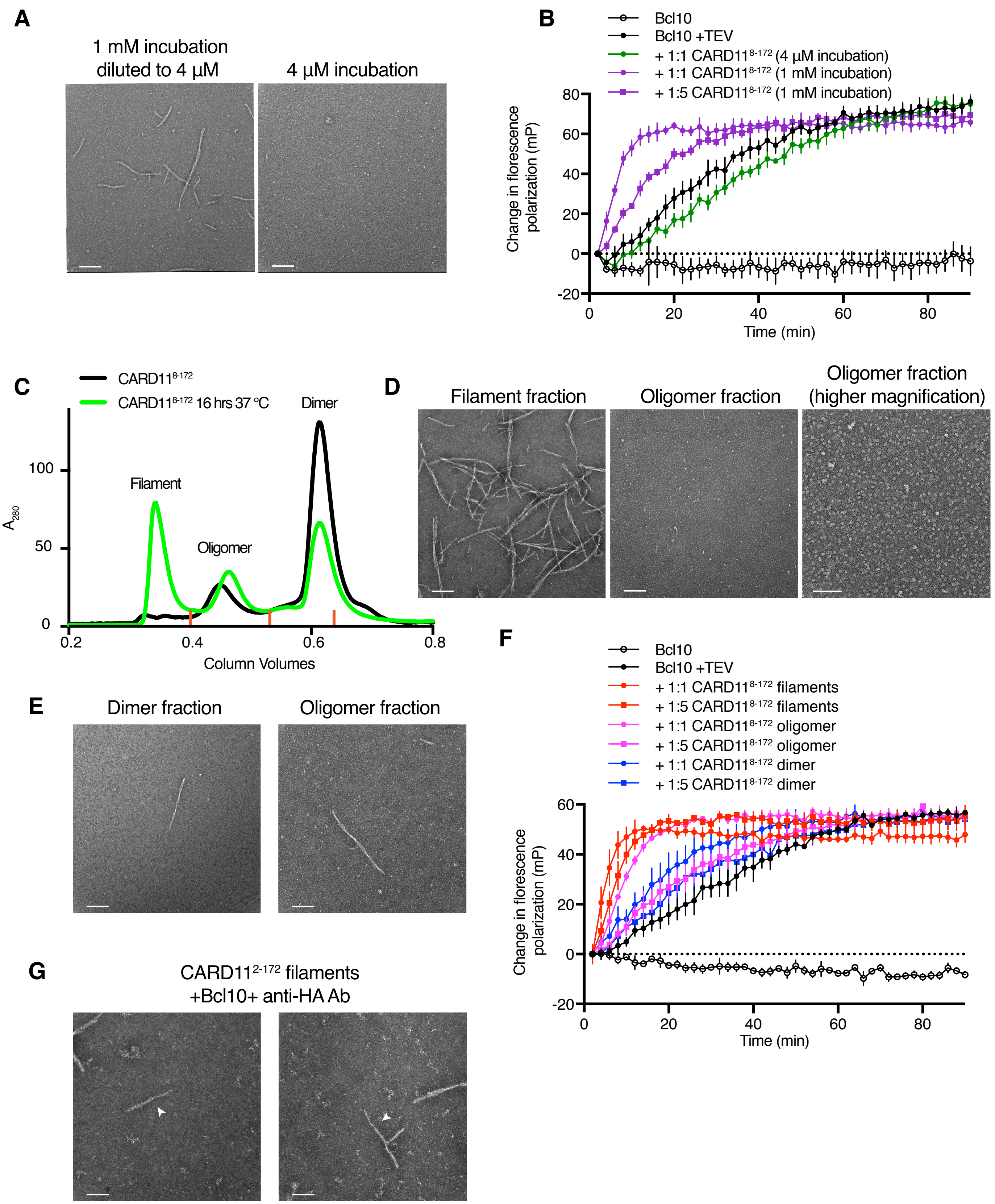
A
1 mM incubation
diluted to 4 µM
4 µM incubation
B
Bcl10
Bcl10 +TEV
+ 1:1 CARD11 8-172 (4 µM incubation)
+ 1:1 CARD11 8-172 (1 mM incubation)
+ 1:5 CARD11 8-172 (1 mM incubation)
Change in florescence polarization (mP)
Time (min)
C
CARD11 8-172
CARD11 8-172 16 hrs 37 °C
Filament
Oligomer
Dimer
A280
Column Volumes
D
Filament fraction
Oligomer fraction
Oligomer fraction (higher magnification)
E
Dimer fraction
Oligomer fraction
F
Bcl10
Bcl10 +TEV
+ 1:1 CARD11 8-172 filaments
+ 1:5 CARD11 8-172 filaments
+ 1:1 CARD11 8-172 oligomer
+ 1:5 CARD11 8-172 oligomer
+ 1:1 CARD11 8-172 dimer
+ 1:5 CARD11 8-172 dimer
Change in florescence polarization (mP)
Time (min)
G
CARD11 2-172 filaments
+Bcl10+ anti-HA Ab

Supplementary Figure 4. CARD11 helical assembly and Bcl10 nucleation, related to Figure 5 $CARD11^{8-172}$ nucleates Bcl10 polymerization only after forming helical assemblies. (A) NS-EM micrographs of $CARD11^{8-172}$ after incubation for 16 hours at 37°C either at 1 mM (left) or 4 μM (right) concentration. Both samples were added to the NS-EM grid at 4 μM. Scale bars are 200 nm. (B) Bcl10 FP polymerization assay. MBP-Bcl10 and $CARD11^{8-172}$ (purple, incubated at 1 mM for 16 hours at 37°C; green, incubated at 4 μM for 16 hours at 37°C) were mixed at the indicated molar ratios. To the indicated samples, TEV protease was added at time t=0. Bcl10 polymerization was monitored by an increase in FP. Vertical bars represent the standard deviation of four technical replicates. (C) Superdex 200 increase size exclusion chromatography A_{280} trace of $CARD11^{2-172}$ after purification and concentration to 1 mM (black) and after subsequent incubation for 16 hours at 37°C (green). Orange lines indicate positions of molecular weight standards of 670 kDa, 158 kDa, and 44 kDa. (D) Representative NS-EM micrographs of SEC fractions from panel C corresponding to the filament peak (left) and the oligomer peak (center, right). Scale bars are 200 nm (left, center) or 100 nm (right) (E) NS-EM micrographs from the dimer and oligomer peaks from the SEC trace in panel C. Filaments were much less prevalent than in the filament fraction, but could be found with minimal searching. Scale bars are 200 nm. (F) Bcl10 FP polymerization assay. $CARD11^{8-172}$ fractions from the SEC trace in panel C were quantified and added at a 1:1 or 1:5 molar ratio to 2 μM Bcl10. TEV protease was added at time t=0, with Bcl10 polymerization monitored by FP. Vertical bars represent the standard deviation of four technical replicates. (G) Nucleation of Bcl10 by $CARD11^{8-172}$ filaments 2 minutes after addition of TEV protease and with anti-HA antibody added during NS-EM grid preparation. White arrows indicate $CARD11^{8-172}$-to-Bcl10-filament transitions. Sample was prepared under identical conditions and concentrations as in panel E (red circles) with grid prepared 2 minutes after addition of TEV protease. See Experimental Methods for details about antibody addition during grid preparation. Scale bars are 200 nm. All source data are provided in Source Data file.

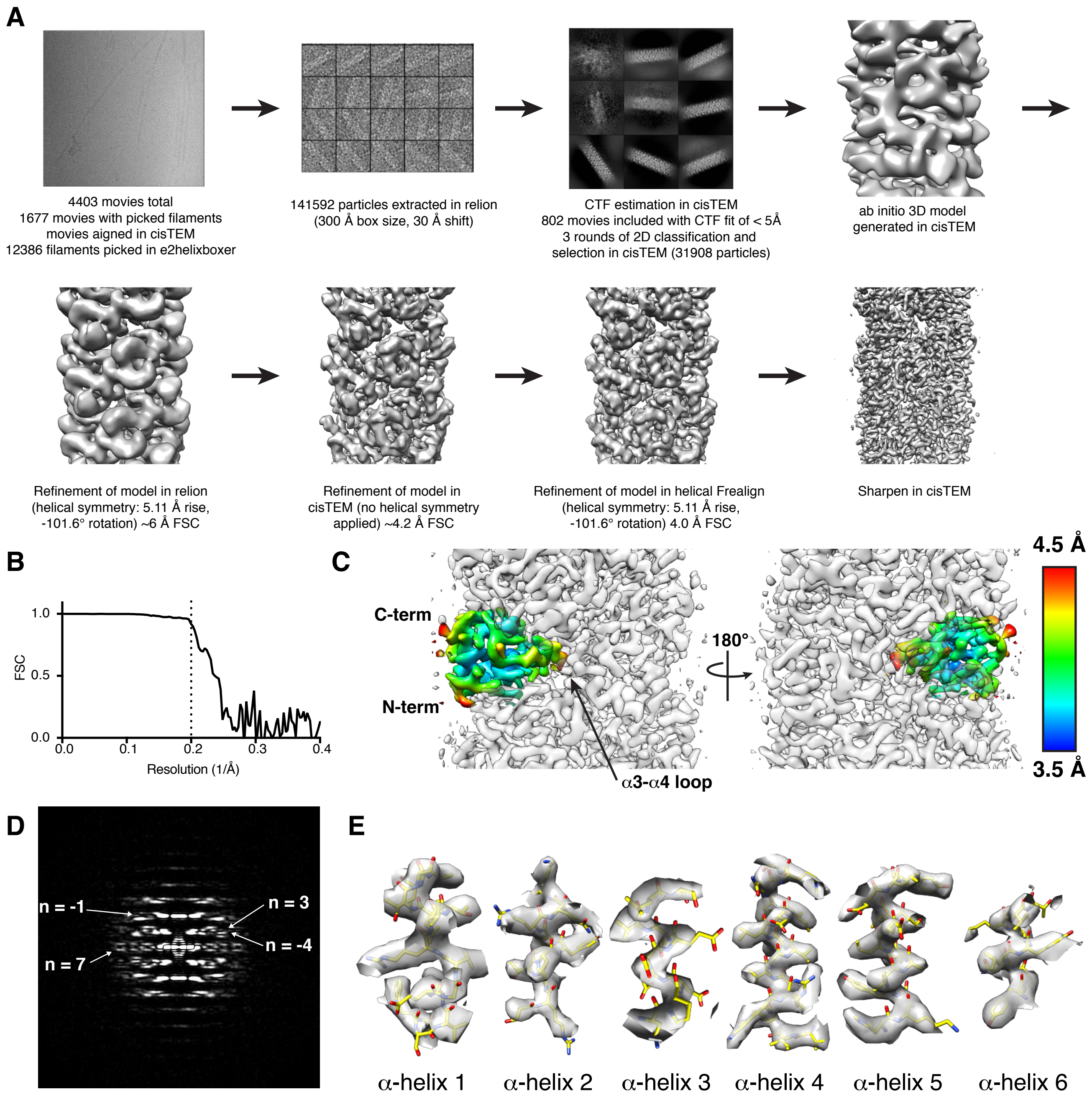

Supplementary Figure 5. Cryo-EM processing, structural characterization, and modeling, related to Figure 6. (A) Visual depiction of the Cryo-EM data processing scheme to generate the CARD9$^{2\text{-}152/I107E}$ structure. See Experimental Methods for detailed description of data processing and refinement. (B) Fourier shell correlation curve for the final CARD9$^{2\text{-}152/I107E}$ structure refined in Frealign. (C) Local resolution estimate for the CARD9$^{2\text{-}152/I107E}$ structure. Local resolution is mapped onto density corresponding to a single CARD, with the remaining density depicted in transparent gray. (D) Power spectrum of a representative class average of the CARD9$^{2\text{-}152/I107E}$ filament. Layer lines corresponding to the prominent 1-, 3-, 4-, and 7-start symmetries are indicated. (E) EM density and modeled structure for each of the 6 helices of the CARD.

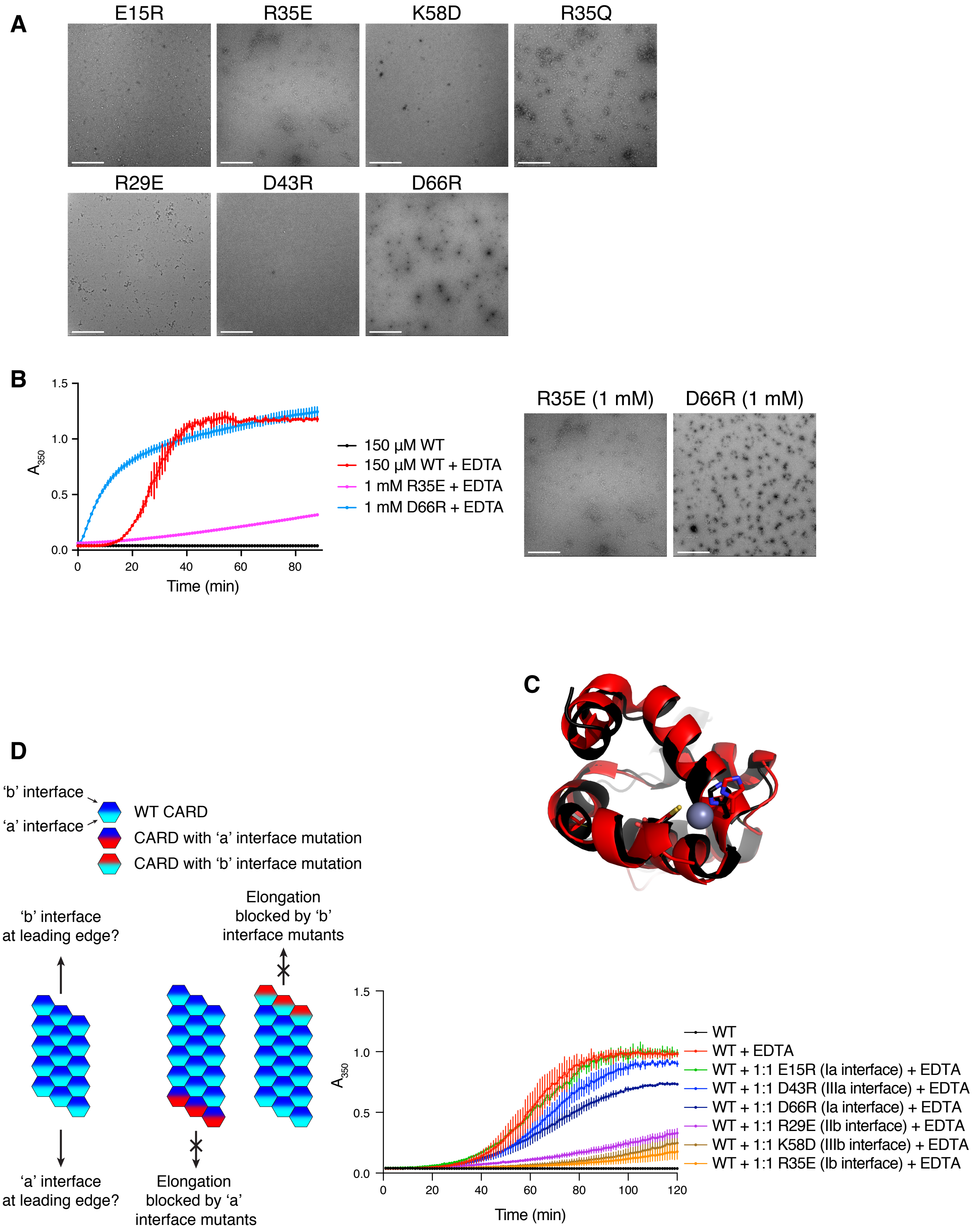
A
E15R
R35E
K58D
R35Q
R29E
D43R
D66R
B
1.5
1.0
0.5
0.0
A350
0
20
40
60
80
Time (min)
150 µM WT
150 µM WT + EDTA
1 mM R35E + EDTA
1 mM D66R + EDTA
R35E (1 mM)
D66R (1 mM)
C
D
'b' interface
'a' interface
WT CARD
CARD with 'a' interface mutation
CARD with 'b' interface mutation
'b' interface at leading edge?
'a' interface at leading edge?
Elongation blocked by 'b' interface mutants
Elongation blocked by 'a' interface mutants
1.5
1.0
0.5
0.0
A350
0
20
40
60
80
100
120
Time (min)
WT
WT + EDTA
WT + 1:1 E15R (Ia interface) + EDTA
WT + 1:1 D43R (IIIa interface) + EDTA
WT + 1:1 D66R (Ia interface) + EDTA
WT + 1:1 R29E (IIb interface) + EDTA
WT + 1:1 K58D (IIIb interface) + EDTA
WT + 1:1 R35E (Ib interface) + EDTA

Supplementary Figure 6. Validation of filament structure and determination of directionality, related to Figure 6. (A) NS-EM micrographs generated from the assay depicted in Figure 6D. Grids were prepared at the endpoint of the assay with no dilution. Scale bars are 1 μm for all panels. (B) (left) $CARD9^{2\text{-}97}$ polymerization assay. At time t=0, a super-stoichiometric concentration of EDTA was added to each CARD, which were pre-saturated with 1:1 Zn^{2+}. Polymerization/aggregation was monitored by absorbance at 350 nm. WT curves are identical data as depicted in Figure 6D. (right) NS-EM micrographs from grids prepared at the assay endpoint with no dilution. Vertical bars represent the standard deviation of three technical replicates. (C) Alignment of the $CARD9^{2\text{-}97}$ Zn^{2+}-bound crystal structure (black, PDBID 6E27) and the Cryo-EM CARD structure (red). Critical coordinating residues Cys10 and His73 are depicted as sticks. The Zn^{2+} ion from the crystal structure is shown as a gray ball. (D) $CARD9^{2\text{-}97}$ polymerization assay with competition from polymerization-incompetent mutants. (left) Cartoon schematic of the two potential directionalities of CARD9 CARD polymerization, with 'a' or 'b' interfaces at the leading edge, and the impact of competition by CARDs containing 'a' or 'b' interface mutants on wild-type CARD polymerization, given each potential directionality. (right) $CARD9^{2\text{-}97/WT}$ was mixed 1:1 with $CARD9^{2\text{-}97}$ mutants (150 μM final concentration for each protein) that are unable to form filaments. The interface affected by each mutant is indicated (see Figure 6C). At time t=0, 400 μM EDTA was added to each sample to strip away all Zn^{2+}. Mutations in 'a' interfaces have minimal impact on $CARD9^{2\text{-}97/WT}$ polymerization while mutations in 'b' interfaces significantly disrupt polymerization, indicating that the CARD9 CARD polymerizes unidirectionally, with the 'b' interface at the leading edge (Figure 6A, top). Vertical bars represent the standard deviation of three technical replicates. All source data are provided in Source Data file

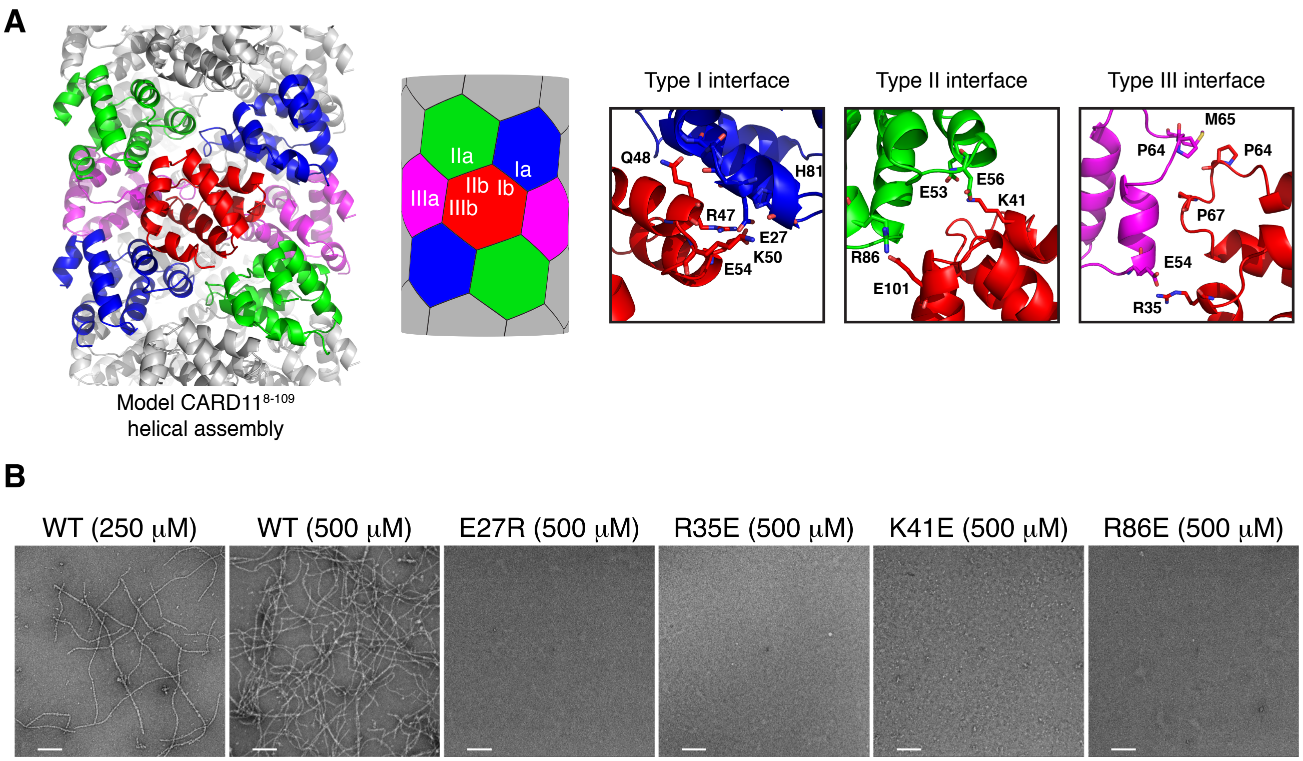

Supplementary Figure 7. Modeling and experimental validation of the CARD11 CARD helical assembly, related to Figure 6. (A) A model of the CARD11 CARD helical filament, generated using Rosetta. The canonical Type I, Type II, and Type III interfaces are depicted, with residues involved in inter-CARD interactions labeled and shown as sticks. (B) NS-EM micrographs of WT or mutant CARD11$^{8-109}$ after a 2 hour incubation at 250 μM or 500 μM as indicated. Scale bars are 200 nm.

Supplementary Table 1. Primers used in this study

Primer name	Sequence
CARD9_pCMV6_C37Y_fwd	GCGGCAGTACAAGGTCCTGAACCCTGATGATGAGGAGCAG
CARD9_pCMV6_C37Y_rev	GACCTTGTACTGCCGCAGGTAAGGTGTGATGCGTGAGGG
CARD9_pCMV6_E81D_fwd	CTTCCTCGATAGCCTGGAGCTCTACTACCCGCAGCTGTAC
CARD9_pCMV6_E81D_rev	CCAGGCTATCGAGGAAGGCCACGTAGCCCTTGTGGC
CARD9_pCMV6_L85Y_fwd	GCCTGGAGTACTACTACCCGCAGCTGTACAAGAAGGTCACAG
CARD9_pCMV6_L85Y_rev	GGTAGTAGTACTCCAGGCTCTCGAGGAAGGCCACGTAGC
CARD9_pCMV6_Y86F_fwd	GGAGCTCTTCTACCCGCAGCTGTACAAGAAGGTCACAGGC
CARD9_pCMV6_Y86F_rev	CGGGTAGAAGAGCTCCAGGCTCTCGAGGAAGGCCAC
CARD9_pCMV6_R101Q_fwd	GCCGGCCCAAGTCTTCTCCATGATCATCGACGCGTCCG
CARD9_pCMV6_R101Q_rev	GAAGACTTGGGCCGGCTCCTTGCCTGTGACCTTCTTGTACAG
CARD9_pCMV6_F103L_fwd	CCCGCGTCTTATCCATGATCATCGACGCGTCCGGGGAG
CARD9_pCMV6_F103L_rev	TCATGGATAAGACGCGGGCCGGCTCCTTGCCTGTGAC
CARD9_pCMV6_I106V_fwd	TCTCCATGGTCATCGACGCGTCCGGGGAGTCAGGCC
CARD9_pCMV6_I106V_rev	CGTCGATGACCATGGAGAAGACGCGGGCCGGCTCC
CARD9_pCMV6_I107E_fwd	CATGATCGAGGACGCGTCCGGGGAGTCAGGCCTGAC
CARD9_pCMV6_I107E_rev	ACGCGTCCTCGATCATGGAGAAGACGCGGGCCGGC
CARD9_pCMV6_G111S_fwd	CGCGTCCTCGGAGTCAGGCCTGACTCAGCTGCTGATG
CARD9_pCMV6_G111S_rev	TGACTCCGAGGACGCGTCGATGATCATGGAGAAGACGCGG
CARD9_pCMV6_G114D_fwd	GGAGTCAGACCTGACTCAGCTGCTGATGACTGAGGTCATGAAG
CARD9_pCMV6_G114D_rev	GAGTCAGGTCTGACTCCCCGGACGCGTCGATGATCATG
CARD9_pCMV6_L115I_fwd	AGTCAGGCATTACTCAGCTGCTGATGACTGAGGTCATGAAGCTG
CARD9_pCMV6_L115I_rev	AGCTGAGTAATGCCTGACTCCCCGGACGCGTCGATGATC
CARD9_pET52_I107E_fwd	CATGATCGAGGATGCCAGCGGCGAGAGTGGTCTGACC
CARD9_pET52_I107E_rev	CTGGCATCCTCGATCATGCTAAAAACACGTGCCGGCTCCTTG
CARD9_pET52_L115I_fwd	AGAGTGGTATCACCCAACTGCTGATGACCGAGGTTATGAAACTG
CARD9_pET52_L115I_rev	AGTTGGGTGATACCACTCTCGCCGCTGGCATCGATGATC
CARD9_pET52_E15R_fwd	CGTTCTGCGGGGTTTTCGTGTGACACTGACAAGTGTGATTGAC
CARD9_pET52_E15R_rev	GAAAACCCCGCAGAACGCTCCAGCACTCGTCGTCGTTTTC
CARD9_pET52_R29D_fwd	ACCCGAGCGATATTACCCCGTACCTGCGTCAGTGCAAAGTG
CARD9_pET52_R29D_rev	GGGTAATATCGCTCGGGTCAATCACACTTGTCAGTGTCACACG
CARD9_pET52_R35E_fwd	GTACCTGGAGCAGTGCAAAGTGCTGAACCCGGATGACGAG
CARD9_pET52_R35E_rev	TGCACTGCTCCAGGTACGGGGTAATACGGCTCGGGTC
CARD9_pET52_R35Q_fwd	CGTACCTGCAACAGTGCAAAGTGCTGAACCCGGATGACGAG
CARD9_pET52_R35Q_rev	TGCACTGTTGCAGGTACGGGGTAATACGGCTCGGGTCAATC
CARD9_pET52_D43R_fwd	GAACCCGCGTGACGAGGAGCAGGTGCTGAGCGACC
CARD9_pET52_D43R_rev	CTCGTCACGCGGGTTCAGCACTTTGCACTGACGCAGGTAC
CARD9_pET52_K58D_fwd	TGATCCGCGATCGCAAAGTGGGTGTTCTGCTGGACATTCTGC
CARD9_pET52_K58D_rev	CTTTGCGATCGCGGATCACTAAATTCGGGTCGCTCAGCACC
CARD9_pET52_D66R_fwd	TTCTGCTGCGCATTCTGCAACGCACAGGTCATAAAGGCTACGTG
CARD9_pET52_D66R_rev	GCAGAATGCGCAGCAGAACACCCACTTTGCGCTTGCGG
CARD11_pET52_E27R_fwd	GAAAATGTTCGGTGCAATCGCCACATGCTGAGCCGCTATATCAAC
CARD11_pET52_E27R_rev	CGATTGCACCGAACATTTTCCCACAGCGCATCTTCTTCATCTTTC
CARD11_pET52_R35E_fwd	ATGCTGAGCGAGTATATCAACCCGGCGAAACTGACCCCGTAC
CARD11_pET52_R35E_rev	GTTGATATACTCGCTCAGCATGTGGCGATTGCACTCAACATTTTCC
CARD11_pET52_K41E_fwd	CCGGCGGAACTGACCCCGTACCTGCGTCAATGCAAAGTG
CARD11_pET52_K41E_rev	GGTCAGTTCCGCCGGGTTGATATAGCGGCTCAGCATGTGG
CARD11_pET52_R86E_fwd	GGGTCAGGAGGGCTACGTGGTTTTCCTGGAGAGCCTGG
CARD11_pET52_R86E_rev	GTAGCCCTCCTGACCCTTGGTGTGCAGAATATCCAGCAGAC

Supplementary Table 2. List of protein sequences used, related to Figure 5 and Supplementary Figure 3

Vector	Plasmid	Insert	Protein sequence
V08 (C-terminal tagged mEos3.1)	rhx1140	CARD9$^{2\text{-}98}$	MSDYENDDECWSVLEGFRVTLTSVIDPS<br>RITPYLRQCKVLNPDDEEQVLSDPNLVIR<br>KRKVGVLLDILQRTGHKGYVAFLESLELY<br>YPQLYKKVTGKE
	rhx2138	Bcl10 Full-length	MSEPTAPSLTEEDLTEVKKDALENLRVYL<br>CEKIIAERHFDHLRAKKILSREDTEEISCR<br>TSSRKRAGKLLDYLQENPKGLDTLVESIR<br>REKTQNFLIQKITDEVLKLRNIKLEHLKGL<br>KCSSCEPFPDGATNNLSRSNSDESNFSE<br>KLRASTVMYHPEGESSTTPFFSTNSSLN<br>LPVLEVGRTENTIFSSTTLPRPGDPGAPP<br>LPPDLQLEEEGTCANSSEMFLPLRSRTV<br>SRQ
	rhx2303	CARD9$^{2\text{-}142}$	MSDYENDDECWSVLEGFRVTLTSVIDPS<br>RITPYLRQCKVLNPDDEEQVLSDPNLVIR<br>KRKVGVLLDILQRTGHKGYVAFLESLELY<br>YPQLYKKVTGKEPARVFSMIIDASGESGL<br>TQLLMTEVMKLQKKVQDLTALLSSKDD
	rhx2304	CARD9$^{2\text{-}142/I107E}$	MSDYENDDECWSVLEGFRVTLTSVIDPS<br>RITPYLRQCKVLNPDDEEQVLSDPNLVIR<br>KRKVGVLLDILQRTGHKGYVAFLESLELY<br>YPQLYKKVTGKEPARVFSMIEDASGESG<br>LTQLLMTEVMKLQKKVQDLTALLSSKDD

Supplementary Table 3. List of yeast strains used for DaMFRET, related to Figure 5 and Supplementary Figure 3

Yeast strain	Background	Genotype	Parent strain	Source
rhy1713	S288C	MATα lyp1Δ can1Δ::STE2pr_SpHIS5 his3Δ1 leu2Δ0 ura3Δ0 met15Δ0 cln3Δ0::GAL1pr_WHI5_hphMX		(Khan et al., 2018)
rhy1734	S288C	MATa lyp1Δ can1Δ::STE2pr_SpHIS5 his3Δ1 leu2Δ0 ura3Δ0 met15Δ0 cln3Δ0::GAL1pr_WHI5_hphMX		(Khan et al., 2018)
rhy1903	S288C	MATa lyp1Δ can1Δ::STE2pr_SpHIS5 his3Δ1 leu2Δ0 ura3Δ0 met15Δ0 cln3Δ0::GAL1pr_WHI5_hphMX hoΔ::natMX_tetO7pr_CaURA3_KlURA3_μNS-mTagBFP2	rhy1734	This study
rhy2068	S288C	MATa lyp1Δ can1Δ::STE2pr_SpHIS5 his3Δ1 leu2Δ0 ura3Δ0 met15Δ0 cln3Δ0::GAL1pr_WHI5_hphMX hoΔ::natMX_tetO7pr_CaURA3_KlURA3_mTagBFP2	rhy1734	This study
rhy2077	S288C	MATa lyp1Δ can1Δ::STE2pr_SpHIS5 his3Δ1 leu2Δ0 ura3Δ0 met15Δ0 cln3Δ0::GAL1pr_WHI5_hphMX hoΔ::natMX_tetO7pr_CARD9$^{2\text{-}98}$-μNS-mTagBFP2	rhy1903	This study
rhy2079	S288C	MATa lyp1Δ can1Δ::STE2pr_SpHIS5 his3Δ1 leu2Δ0 ura3Δ0 met15Δ0 cln3Δ0::GAL1pr_WHI5_hphMX hoΔ::natMX_tetO7pr_Bcl10-μNS-mTagBFP2	rhy1903	This study
rhy2089	S288C	MATa lyp1Δ can1Δ::STE2pr_SpHIS5 his3Δ1 leu2Δ0 ura3Δ0 met15Δ0 cln3Δ0::GAL1pr_WHI5_hphMX hoΔ::natMX_tetO7pr_CARD9$^{2\text{-}98}$-mTagBFP2	rhy2068	This study
rhy2228	S288C	MATa lyp1Δ can1Δ::STE2pr_SpHIS5 his3Δ1 leu2Δ0 ura3Δ0 met15Δ0 cln3Δ0::GAL1pr_WHI5_hphMX hoΔ::natMX_tetO7pr_CARD9$^{2\text{-}142}$-mTagBFP2	rhy2068	This study
rhy2229	S288C	MATa lyp1Δ can1Δ::STE2pr_SpHIS5 his3Δ1 leu2Δ0 ura3Δ0 met15Δ0 cln3Δ0::GAL1pr_WHI5_hphMX hoΔ::natMX_tetO7pr_CARD9$^{2\text{-}142/I107E}$-mTagBFP2	rhy2068	This study
rhy2254	S288C	MATa lyp1Δ can1Δ::STE2pr_SpHIS5 his3Δ1 leu2Δ0 ura3Δ0 met15Δ0 cln3Δ0::GAL1pr_WHI5_hphMX hoΔ::natMX_tetO7pr_CARD9$^{2\text{-}142}$-μNS-mTagBFP2	rhy1903	This study
rhy2255	S288C	MATa lyp1Δ can1Δ::STE2pr_SpHIS5 his3Δ1 leu2Δ0 ura3Δ0 met15Δ0 cln3Δ0::GAL1pr_WHI5_hphMX hoΔ::natMX_tetO7pr_CARD9$^{2\text{-}142/I107E}$-μNS-mTagBFP2	rhy1903	This study
