# Supplementary material for: Structures of autoinhibited and polymerized forms of CARD9 reveal mechanisms of CARD9 and CARD11 activation
Source: Nat Commun. 2019 Jul 11;10:3070. doi: 10.1038/s41467-019-10953-z (PMC6624267; doi:10.1038/s41467-019-10953-z)
Supplement: Supplementary file 3 — Reporting Summary [file 41467_2019_10953_MOESM3_ESM.pdf]

nature research

Corresponding author(s): Wayne Fairbrother, Erin Dueber, Alexis Rohou

Last updated by author(s): May 21, 2019

Reporting Summary

Nature Research wishes to improve the reproducibility of the work that we publish. This form provides structure for consistency and transparency in reporting. For further information on Nature Research policies, see Authors & Referees and the Editorial Policy Checklist.

Statistics

For all statistical analyses, confirm that the following items are present in the figure legend, table legend, main text, or Methods section.

n/a	Confirmed	
☐	☒	The exact sample size (n) for each experimental group/condition, given as a discrete number and unit of measurement
☐	☒	A statement on whether measurements were taken from distinct samples or whether the same sample was measured repeatedly
☐	☒	The statistical test(s) used AND whether they are one- or two-sided<br>*Only common tests should be described solely by name; describe more complex techniques in the Methods section.*
☒	☐	A description of all covariates tested
☒	☐	A description of any assumptions or corrections, such as tests of normality and adjustment for multiple comparisons
☒	☐	A full description of the statistical parameters including central tendency (e.g. means) or other basic estimates (e.g. regression coefficient) AND variation (e.g. standard deviation) or associated estimates of uncertainty (e.g. confidence intervals)
☐	☒	For null hypothesis testing, the test statistic (e.g. F, t, r) with confidence intervals, effect sizes, degrees of freedom and P value noted<br>*Give P values as exact values whenever suitable.*
☒	☐	For Bayesian analysis, information on the choice of priors and Markov chain Monte Carlo settings
☒	☐	For hierarchical and complex designs, identification of the appropriate level for tests and full reporting of outcomes
☒	☐	Estimates of effect sizes (e.g. Cohen's d, Pearson's r), indicating how they were calculated

Our web collection on statistics for biologists contains articles on many of the points above.

Software and code

Policy information about availability of computer code

Data collection	TopSpin 3.5, SoftMax Pro 7, SerialEM 3.6, Everest V1.1, Xcaliber
Data analysis	ccpn Analysis 2.4, CYANA 3.97, CNS 1.2, WaterRefCNS, PROCHECK-NMR, PDBePISA, Pymol 1.7, Prism 7, ExMS, cisTEM, EMAN2, RELION 2.1, Frealign 9.11, UCSF Chimera 1.11, Phenix 1.12, COOT 0.8.6, FCS Express Plus 6.04.0015, MASACOT, MATLAB, custom python scripts as described in doi: 10.1073/pnas.1319482110, custom awk scripts to convert between EM data processing platform formats, a re-implementation of blocres (Rohou, manuscript in preparation)

For manuscripts utilizing custom algorithms or software that are central to the research but not yet described in published literature, software must be made available to editors/reviewers. We strongly encourage code deposition in a community repository (e.g. GitHub). See the Nature Research guidelines for submitting code & software for further information.

Data

Policy information about availability of data

All manuscripts must include a data availability statement. This statement should provide the following information, where applicable:

- Accession codes, unique identifiers, or web links for publicly available datasets
- A list of figures that have associated raw data
- A description of any restrictions on data availability

Chemical shifts for the CARD9(2-142) dimer were deposited in the Biological Magnetic Resonance Database (http://www.bmrb.wisc.edu/) under BMRB # 30543. An electron microscopy density map for the CARD9(2-142/I107E) filament has been deposited in the Electron Microscopy Data Bank (https://www.ebi.ac.uk/pdbe/emdb/) under EMDB # 9332. Atomic coordinates of the NMR solution structure of CARD9(2-142) dimer were deposited in the Research Collaboratory for Structural Bioinformatics Protein Databank (https://www.rcsb.org/) under PDB # 6N2M. Atomic coordinates of a representative array of CARD9(2-152/I107E) helical assembly were deposited in the Research Collaboratory for Structural Bioinformatics Protein Databank under PDB # 6N2P. The source data underlying Figures 2C, 3B, 4B, 4F, 4H, 5C, 6D, and Supplementary Figures 2F, 3B, 3E, 4B, 4F, 6B, and 6D are provided as a Source Data file.

Field-specific reporting

Please select the one below that is the best fit for your research. If you are not sure, read the appropriate sections before making your selection.

☒ Life sciences ☐ Behavioural & social sciences ☐ Ecological, evolutionary & environmental sciences

For a reference copy of the document with all sections, see nature.com/documents/nr-reporting-summary-flat.pdf

Life sciences study design

All studies must disclose on these points even when the disclosure is negative.

Sample size	Three biological replicates were determined to be representative of variability in the NF-kB signaling assays.
Data exclusions	No data were excluded
Replication	In-cell NF-kB data were generated with three independent biological replicates, polymerization data were generated using three technical replicates.
Randomization	No randomization was used in this study.
Blinding	No blinding was used in this study.

Reporting for specific materials, systems and methods

We require information from authors about some types of materials, experimental systems and methods used in many studies. Here, indicate whether each material, system or method listed is relevant to your study. If you are not sure if a list item applies to your research, read the appropriate section before selecting a response.

Materials & experimental systems

n/a	Involved in the study	
☐	☒	Antibodies
☐	☒	Eukaryotic cell lines
☒	☐	Palaeontology
☒	☐	Animals and other organisms
☒	☐	Human research participants
☒	☐	Clinical data

Methods

n/a	Involved in the study	
☒	☐	ChIP-seq
☐	☒	Flow cytometry
☒	☐	MRI-based neuroimaging

Antibodies

Antibodies used	anti-human CARD9 (ThermoFisher PA5-19993)<br>anti-human CARD11 (Cell Signaling 4440S)<br>anti-human beta-actin (Licor 926-42212)<br>anti-HA peptide antibody (Sigma H3663)
Validation	All antibodies were commercially generated and validated

Eukaryotic cell lines

Policy information about cell lines

Cell line source(s)	hkb-hnod2, InvivoGen
Authentication	Cell lines were authenticated by InvivoGen
Mycoplasma contamination	Cell line tested negative for mycoplasma contamination
Commonly misidentified lines<br>(See ICLAC register)	*Name any commonly misidentified cell lines used in the study and provide a rationale for their use.*

Flow Cytometry

Plots

Confirm that:

☐ The axis labels state the marker and fluorochrome used (e.g. CD4-FITC).

☒ The axis scales are clearly visible. Include numbers along axes only for bottom left plot of group (a 'group' is an analysis of identical markers).

☒ All plots are contour plots with outliers or pseudocolor plots.

☐ A numerical value for number of cells or percentage (with statistics) is provided.

Methodology

Sample preparation	All flow experiments utilized cultured S. cerevisiae
Instrument	ImageStreamx MkII (Amnis)
Software	FCS Express Plus 6.04.0015 software (De Novo)
Cell population abundance	*Describe the abundance of the relevant cell populations within post-sort fractions, providing details on the purity of the samples and how it was determined.*
Gating strategy	Events were gated for single unbudded cells by FSC vs SSC, followed by gating of live cells with low autofluorescence and donor positive. Live gate was then selected for double positives (donor and acceptor).

☐ Tick this box to confirm that a figure exemplifying the gating strategy is provided in the Supplementary Information.
